# The novel lncRNA GPC5-AS1 stabilizes GPC5 mRNA by competitively binding with miR-93/106a to suppress gastric cancer cell proliferation

**DOI:** 10.18632/aging.203901

**Published:** 2022-02-18

**Authors:** Guo Bo, Yijie Liu, Wen Li, Lumin Wang, Lingyu Zhao, Dongdong Tong, Lei Ni, Liying Liu, Yannan Qin, Wenjing Wang, Chen Huang

**Affiliations:** 1 Department of Cell Biology and Genetics, Key Laboratory of Environment and Genes Related to Diseases, School of Basic Medical Sciences, Xi’an Jiaotong University Health Science Center, Xi’an, P.R. China; 2 Institute of Genetics and Developmental Biology, Translational Medicine Institute, School of Basic Medical Sciences, Xi’an Jiaotong University Health Science Center, Xi’an, P.R. China; 3 Department of Gastroenterology, The Second Affiliated Hospital of Xi’an Jiaotong University, Xi’an, P.R. China; 4 Key Laboratory of Environment and Genes Related to Diseases, Xi’an Jiaotong University, Ministry of Education of China, Xi’an, P.R. China; 5 Department of Hepatobiliary Surgery, The First Affiliated Hospital of Xi’an Jiaotong University, Xi’an, P.R. China; 6 Key Laboratory of Shaanxi Province for Craniofacial Precision Medicine Research, Xi’an, P.R. China

**Keywords:** gastric cancer, long non-coding RNA, methyl-CpG binding protein 2, competing endogenous RNAs, molecular sponge

## Abstract

Long non-coding RNAs (lncRNAs) are of importance in the genesis and progression of gastric cancer (GC). GPC5-AS1 is a novel lncRNA associated with methyl-CpG-binding protein 2 (MeCP2), identified in our previous microarray analysis; however, the role of GPC5-AS1 in GC remains unknown. In the present study, we demonstrate that GPC5-AS1 is downregulated in GC cells and tissues, and this aberrant expression is regulated by MeCP2 through CpG site binding in the promoter region. Importantly, we also demonstrate that GPC5-AS1 overexpression suppresses cell proliferation, colony formation, and cell cycle transition; induces apoptosis *in vitro*; and inhibits tumorigenicity *in vivo*. The expression of the controversial gene *GPC5* was downregulated in GC tissues, and elevated GPC5 level could inhibit GC cell growth. Mechanistically, we demonstrated that GPC5-AS1 stabilizes GPC5 mRNA by acting as a molecular sponge for miR-93 and miR-106a, thereby reducing GC tumor progression. In conclusion, our results suggest that GPC5-AS1 may play a pivotal role in GC and serve as a potential diagnostic biomarker and a powerful therapeutic target for GC.

## INTRODUCTION

Gastric cancer (GC) is one of the most frequently occurring malignancies and remains a serious risk to human health in the world [1]. Owing to the lack of typical clinical features in the early phase and the high failure rate of chemotherapy, patients with GC are often diagnosed in the advanced stage and have a poor prognosis. Novel therapeutic strategies to improve patient outcome are urgently needed. In general, GC formation and progression involve multiple steps that include various gene mutations or genomic alterations. Elucidating the exact molecular mechanisms of GC progression is crucial for developing effective treatments [2].

Long non-coding RNAs (lncRNAs) are a class of non-protein coding transcripts longer than 200 nt and considered a key regulatory molecule in physiological processes including chromatin modification, transcription or translation, cell differentiation, cell cycle regulation, and oncogenic or antineoplastic signaling in cancer [3]. Accumulating evidence indicates that lncRNAs play complex and a wide range of roles in the occurrence and development of GC [4]. LncRNA HOTAIR, for instance, has been found to be overexpressed in GC, acting as a biomarker for poor prognosis. The ectopic expression of HOTAIR promotes malignant behavior by regulating human epithelial growth factor receptor 2 (HER2) expression in GC cells [5]. In addition, the lncRNA HOXA11-AS is reportedly a key effector of GC genesis and progression via a crosstalk between EZH2/HOXA11-AS/LSD1 and HOXA11-AS/miR-1297/EZH2 [6]. Methyl-CpG-binding protein 2 (MeCP2) is one of the methyl-CpG-binding domain (MBD) family members, acting as a crucial epigenetic factor in the regulation of gene transcription and gene promoter activity by binding to methylated DNA [7–10]. In our previous study, microarray analysis indicated that the novel lncRNA GPC5-AS1 may be a direct target of MeCP2. MeCP2 facilitates GC cell growth and restrains cell apoptosis by regulating FOXF1/MYOD1 and GIT1 transcription through binding to the methylated CpG islands in the promoter [11]. However, the role of GPC5-AS1 in GC remains unknown.

Through a newly identified post-transcriptional regulatory mechanism of action, lncRNAs have been shown to interact with target RNAs directly or indirectly, thereby affecting RNA production [12, 13]. Acting as competing endogenous RNAs (ceRNAs), accumulating evidence suggests that lncRNAs can competitively bind to a number of microRNAs (miRNAs) and shield their target mRNAs. Both lncRNAs and miRNAs often interact to achieve biological functions in several physiological or pathological processes in carcinogenesis [14, 15]. For instance, SNHG12 is significantly overexpressed in GC and promotes tumorigenesis by directly targeting miR-320 [16]. The lncRNA BC032469 is a ceRNA of miR-1207-5p that can upregulate the expression of hTERT and promote cell growth in GC [17]. In contrast, the downregulation of the lncRNA MT1JP is related with the phenotypes of malignant tumor and the survival of patients with GC; it acts as a ceRNA by binding to miR-92a-3p and regulating the expression of FBXW7 [18]. The biological function of GPC5-AS1 and its potential mechanisms in GC progression have not been fully elucidated.

In this study, we used qRT-PCR to identify whether MeCP2 regulates the differential expression of GPC5-AS1 in GC tissues and normal tissues. The gain of function of GPC5-AS1 suppressed GC cell proliferation, induced cell apoptosis *in vitro*, and inhibited xenograft formation in a mouse model. Importantly, we confirmed that GPC5-AS1 is a miRNA sponge by sequestering miR-93 and miR-106a and stabilizing GPC5 mRNA, thus playing a facilitative role in GC tumorigenesis. Our findings indicate that GPC5-AS1 holds potential as an innovative diagnostic biomarker and an accessible therapeutic target for GC.

## RESULTS

### Aberrant GPC5-AS1 expression is observed in GC

**Figure 1:**
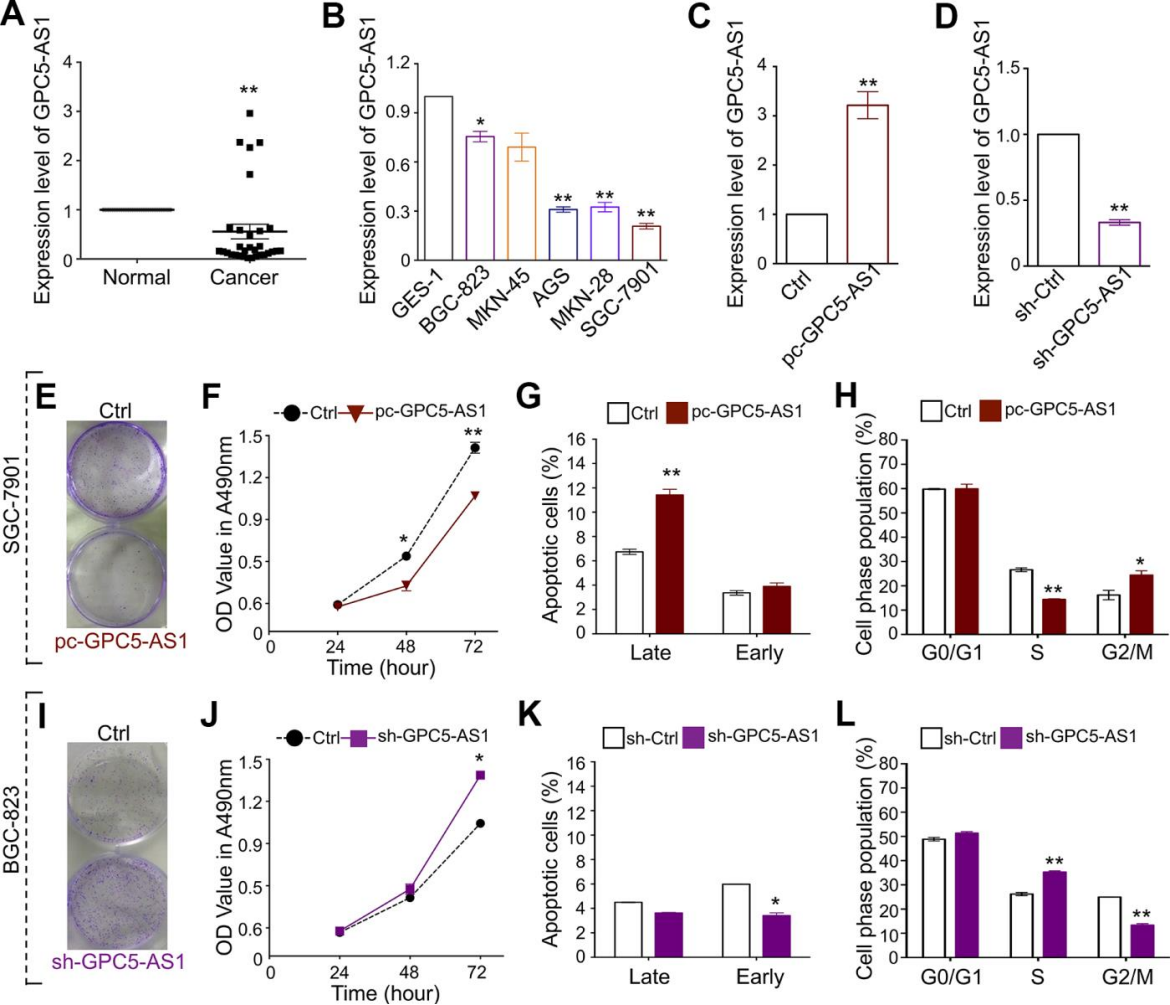
**GPC5-AS1 is down-regulated in GC and overexpression of GPC5-AS1 inhibits cell proliferation *in vitro*.** (**A**, **B**) The expression of GPC5-AS1 in GC tissue samples and cell lines was measured by qRT-PCR. (**C**) Measurement of GPC5-AS1 expression level in SGC-7901 cells with pc-GPC5-AS1. (**D**) Measurement of GPC5-AS1 expression level in BGC-823 cells with indicated shRNA. (**E**) Colony formation, (**F**) MTT assay, (**G**) cell apoptosis, and (**H**) cell cycle assay were performed in SGC-7901 cells with pc-GPC5-AS1. (**I**–**L**) Cell function analyses were determined in BGC-823 cells with sh-GPC5-AS1. (p* < 0.05, p** < 0.01).

To investigate the effect of GPC5-AS1 in GC, matched carcinoma tissues and normal tissues (31 pairs) were obtained and qRT-PCR was performed. GPC5-AS1 expression was considerably downregulated in GC tissues compared with that in normal tissues (Figure 1A). Furthermore, the expression of GPC5-AS1 was significantly lower in four GC cell lines (BGC-823, SGC-7901, MKN-28, and AGS) than that in normal gastric epithelial cell line (GES-1) (Figure 1B). These findings suggest that GPC5-AS1 is dysregulated in GC, and this reduction may be involved in the progression of GC.

### GPC5-AS1 overexpression inhibits GC cell proliferation *in vitro*

Based on the GPC5-AS1 expression in GC cell lines, gain-of-function and loss-of-function studies have been performed to determine the role of GPC5-AS1 in SGC-7901 and BGC-823 cells, respectively. The qRT-PCR analysis demonstrated satisfactory overexpression or downregulation of GPC5-AS1 using a GPC5-AS1 expression plasmid or short hairpin RNA (shRNA) targeting GPC5-AS1, respectively (Figure 1C, 1D). Subsequently, MTT and colony forming assays, cell cycle analyses, and cell apoptosis analyses were performed. The results showed that the enforced expression of GPC5-AS1 led to a reduction in colony formation (Figure 1E) and cell growth at both 48 and 74 h post transfection (Figure 1F), high apoptotic rates in the late phase (Figure 1G), marker accumulation in the G2-phase, and a decrease in S-phase population of SGC-7901 cells (Figure 1H). Furthermore, silencing GPC5-AS1 with shRNA showed that a reduction in GPC5-AS1 could contribute to the tumorigenicity of BGC-823 cells. Transfection with sh-GPC5-AS1 promoted colony formation (Figure 1I), induced cell growth (Figure 1J), moderately affected cell apoptosis (Figure 1K), and blocked S-G2 transition (Figure 1L). The results suggest the contribution of endogenous GPC5-AS1 in the inhibition of GC progression.

### GPC5-AS1 suppresses the growth of GC cells *in vivo*

**Figure 2:**
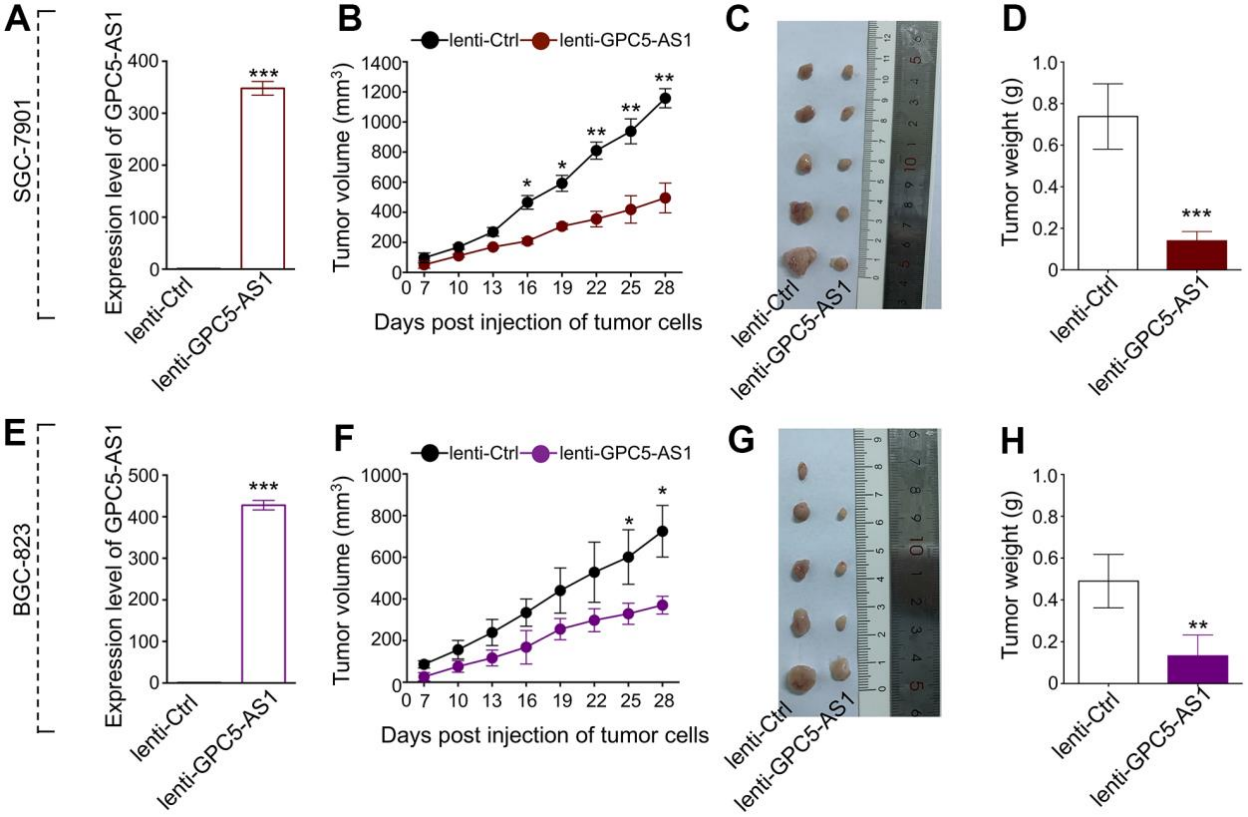
**Enforced expression of GPC5-AS1 attenuated tumor growth *in vivo*.** (**A**) GPC5-AS1 expression was detected by qRT-PCR in SGC-7901 cells transfected with lenti-GPC5-AS1 compared with lenti-ctrl. (**B**) Tumor volume of xenograft tumors of mice inoculated with GPC5-AS1 stably expressing SGC-7901 cells was recorded once every three days. (**C**) Tumor images and (**D**) weight on the day mice euthanized were obtained and present. (**E**–**H**) The anti-tumor ability of GPC5-AS1 was verified in BGC-823 cells transfected with lenti-GPC5-AS1. (n=5, p* < 0.05, p** < 0.01, p*** < 0.001).

To predict the biological function of GPC5-AS1 in GC progression, we explored the proliferative potential of GPC5-AS1 using an *in vivo* tumor xenograft model. The GPC5-AS1 level in SGC-7901 or BGC-823 cells transfected with lenti-GPC5-AS1 was assessed using qRT-PCR to ensure stable overexpression in comparison with that in lenti-ctrl-transfected cells (Figure 2A, 2E). The cells were then injected into each side of the flank of nude mice, and tumor size was monitored every 3 days for 4 weeks. Growth curves demonstrated that GPC5-AS1 significantly reduced tumor growth in mice, regardless of the cancer cell line (Figure 2B, 2F). At the end of the *in vivo* experiment, all mice were humanely euthanized for tumor excision and weighing (Figure 2C, 2G). On an average, the mass of SGC-7901 cells that formed tumors in the lenti-GPC5-AS1 transfected SGC-7901 treatment group was only 19.0 % of the total tumor weight in the control (Figure 2D), whereas the mass of BGC-823 cells that formed tumors in the lenti-GPC5-AS1 transfected BGC-823 treatment group was approximately 27.1 % of the total tumor weight in the control (Figure 2H). These data indicate that GPC5-AS1 could remarkably inhibit the tumorigenicity of GC cells in a nude mouse xenograft model.

### MeCP2 regulates the expression of GPC5-AS1 by binding to its promoter

**Figure 3:**
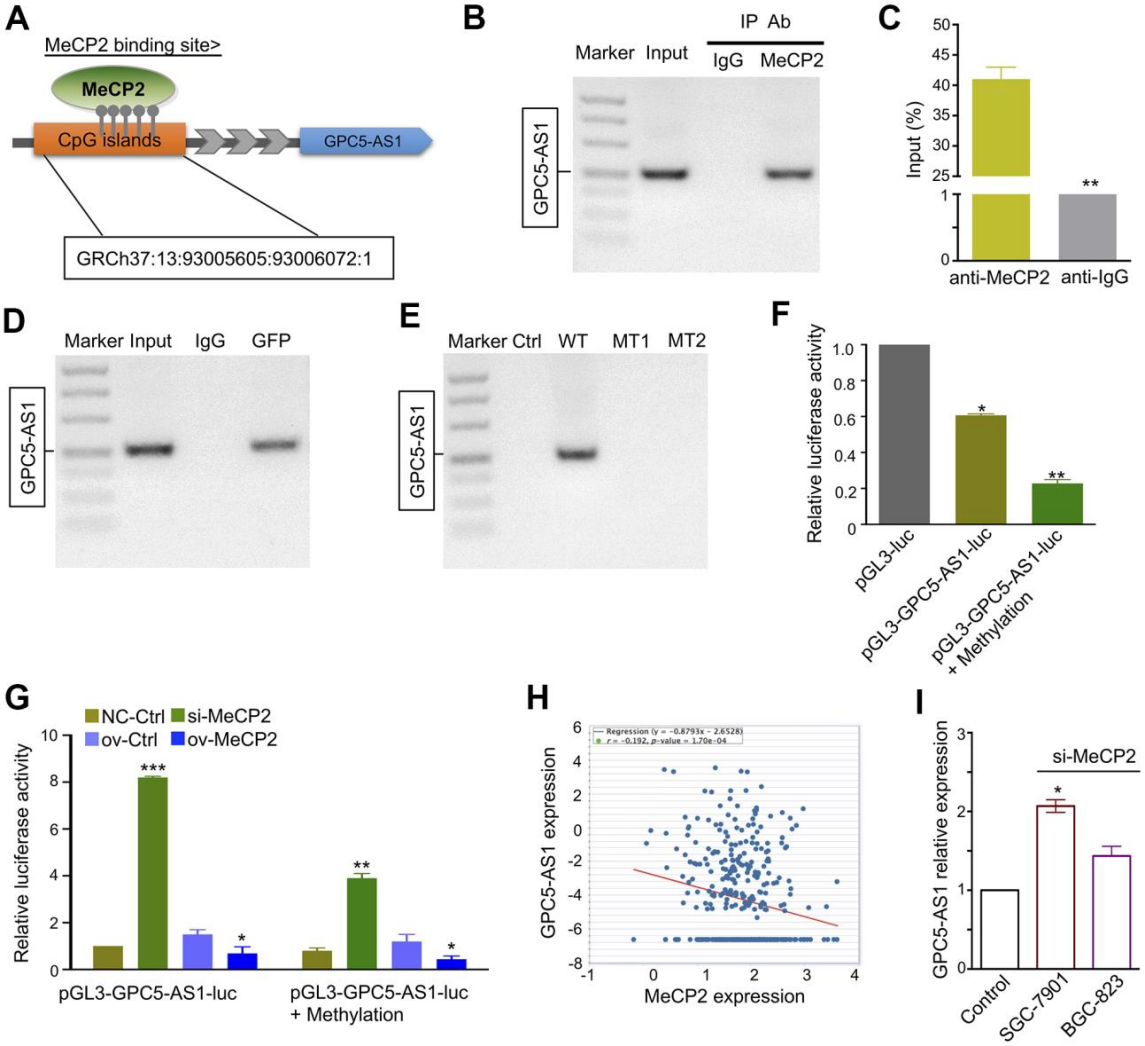
**MeCP2 regulates GPC5-AS1 expression by binding its promoter regions.** (**A**) MeCP2 binding sites were relative to the CpG island location. (**B**, **C**) ChIP RT-PCR of GPC5-AS1 was performed with an anti-MeCP2 antibody. (**D**) ChIP RT-PCR of GPC5-AS1 was performed with an anti-GFP antibody after transfection with the GFP-MeCP2 plasmid. (**E**) ChIP RT-PCR of GPC5-AS1 was performed with an anti-GFP antibody after transfection with Ctrl (GFP plasmid), WT (GFP-MeCP2 plasmid), MT1 (GFP-Mutation type 1), and MT2 (Mutation type 2). (**F**) SGC-7901 cells were transfected with pGL3-GPC5-AS1-luc and pGL3-GPC5-AS1-luc + Methylation; luciferase activity was determined at 48 h post-transfection. Renilla luciferase served as the internal control. (**G**) SGC-7901 cells were treated with pGL3- GPC5-AS1-luc, pGL3-GPC5-AS1-luc + Methylation, MeCP2 siRNAs, and overexpression vectors; luciferase activity was determined. (**H**) Correlation analysis of MeCP2 and GPC5-AS1 expression in GC tissues through starBase (r = -0.192, *p* < 0.05). (**I**) Expression of GPC5-AS1 in SGC-7901 and BGC-823 cells transfected with si-MeCP2. (p* < 0.05, p** < 0.01, p*** < 0.001).

GPC5-AS1 was first identified by chromatin immunoprecipitation sequencing (ChIP-Seq) in our previous study [19]. It contains a CpG site within the promoter regions that may interact with MeCP2 (Figure 3A). ChIP-RT-PCR confirmed that MeCP2 could directly bind to the promoters of GPC5-AS1 in SGC-7901 cells (Figure 3B, 3C). After transfecting SGC-7901 cells with WT or MT GFP-MeCP2 plasmids, we used ChIP-RT-PCR with an anti-GFP antibody and found that exogenous MeCP2 could bind with the CpG sites on GPC5-AS1 promoter regions (Figure 3D). In addition, although GFP-WT also bound to the CpG sites of GPC5-AS1, GFP plasmid (Ctrl) or GFP-MT (1 and 2) did not (Figure 3E). Subsequently, promoter reporter assay was performed to determine whether MeCP2 binds with the CpG sites on the GPC5-AS1 promoter. The sequence of binding sites from previous ChIP-Seq data was inserted into the upstream of luciferase gene in the pGL3 reporter vector. The luciferase signals of SGC-7901 cells was detected at 48 h after transfection, and the results indicated that the luciferase activity of both pGL3-GPC5-AS1-luc and pGL3-GPC5-AS1-luc+methylation groups were considerably reduced compared with that of the pGL3 group (Figure 3F). After transfection with pGL3-GPC5-AS1-luc, the luciferase activity of the MeCP2 siRNA group was increased compare with that of the NC siRNA group, whereas the luciferase activity of the MeCP2 overexpression group was reduced compared with that of the control. Transfection with the pGL3-GPC5-AS1-luc+methylation vector provided similar results, and the luciferase activity of the GPC5-AS1-luc+methylation group was reduced compared with that of the pGL3-GPC5-AS1-luc group (Figure 3G). These data confirm that MeCP2 is a critical regulator of GPC5-AS1 expression in GC cells. To further verify this finding, we examined GPC5-AS1 expression in TCGA database. The mRNA expression of MeCP2 was significantly positively correlated with GPC5-AS1 expression in human GC tissues (Figure 3H), and GPC5-AS1 expression was upregulated after silencing MeCP2 with si-MeCP2, especially in SGC-7901 cells (Figure 3I).

### GPC5 suppresses GC cell proliferation and induces apoptosis

**Figure 4:**
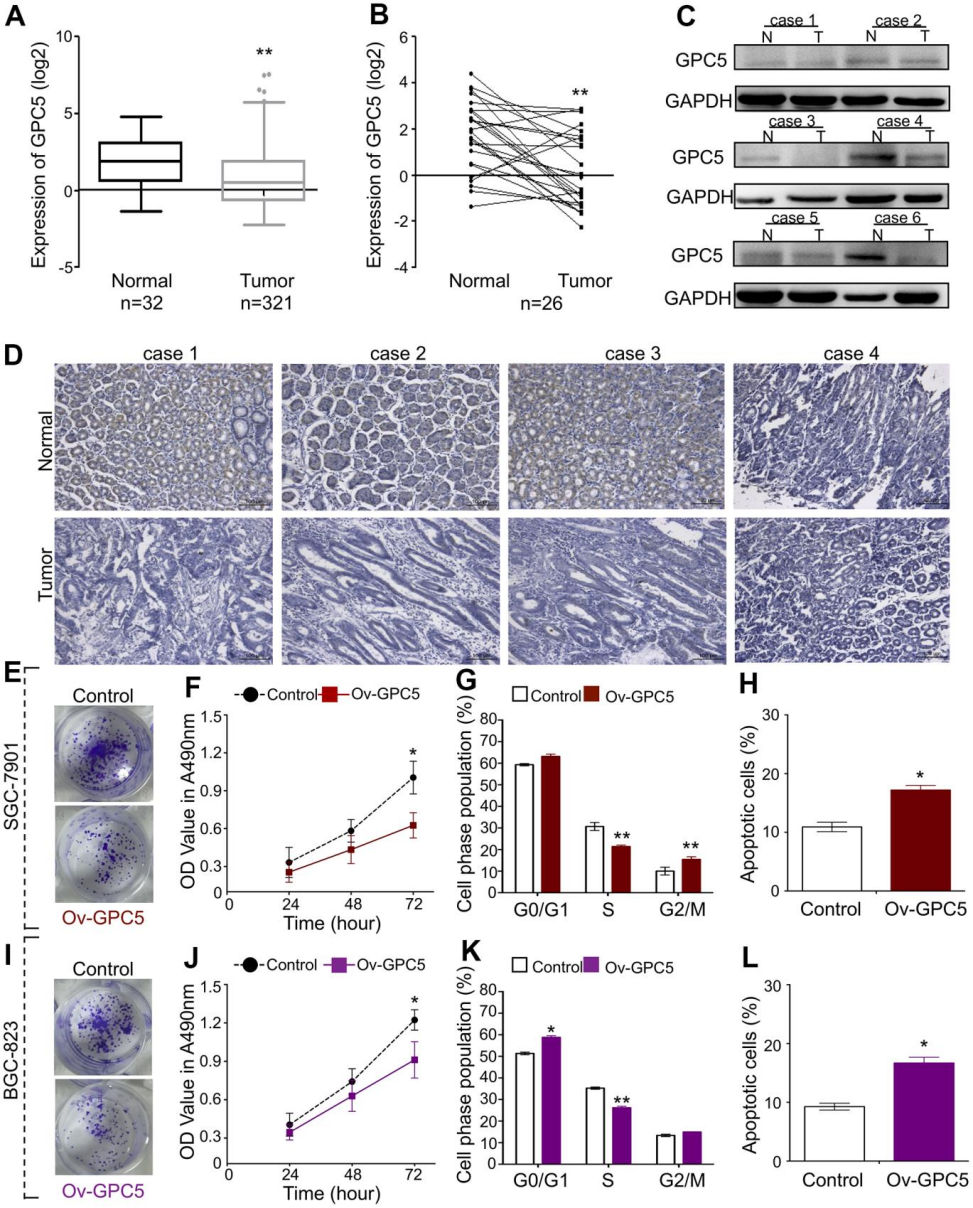
**GPC5 suppresses GC cell proliferation and induces apoptosis.** (**A**, **B**) Expression levels of GPC5 in GC tissues and their paired normal tissues according to the TCGA database. (**C**, **D**) Protein expression of GPC5 was measured by western blot and immunohistochemistry in randomly selected GC tissue samples. (**E**) Colony formation, (**F**) MTT assay, (**G**) cell cycle, and (**H**) cell apoptosis assay were performed in SGC-7901 cells with Ov-GPC5. (**I**–**L**) Cell function analyses were determined in BGC-823 cells with Ov-GPC5. (p* < 0.05, p** < 0.01).

To examine the function of GPC5, based on the sequencing data from the starBase database, we found that GPC5 mRNA expression was downregulated in 321 non-paired and 26 paired tumor tissues compared with that in matched normal tissues (Figure 4A, 4B). The protein expression level of GPC5 was analyzed in randomly selected tissues using western blotting and immunohistochemistry, and was found to be significantly decreased in GC tissues (Figure 4C, 4D). Using an overexpression plasmid, the cellular effects of GPC5 on both BGC-823 and SGC-7901 cells were studied *in vitro*. Colony formation and MTT assays demonstrated that enforced GPC5 expression in GC cells can greatly inhibit cell proliferation (Figure 4E, 4F, 4I, 4J). Cell cycle and apoptosis of GC cells were analyzed using flow cytometry after transfection with ov-GPC5. The introduction of ov-GPC5 resulted in a remarkable decrease in the S-phase of both GC cell lines, a significant accumulation in G2/M in SGC-7901 cells (Figure 4G), and a slight cell cycle arrest in G1-S transition in BGC-823 cells (Figure 4K). Meanwhile, significantly elevated rates of apoptosis were observed due to the overexpression of GPC5 in both BGC-823 and SGC-7901 cells (Figure 4H, 4L). Overall, these findings reveal that GPC5 might be a tumor suppressor in GC progression.

### GPC5-AS1 regulates GPC5 expression by enhancing mRNA stability

**Figure 5:**
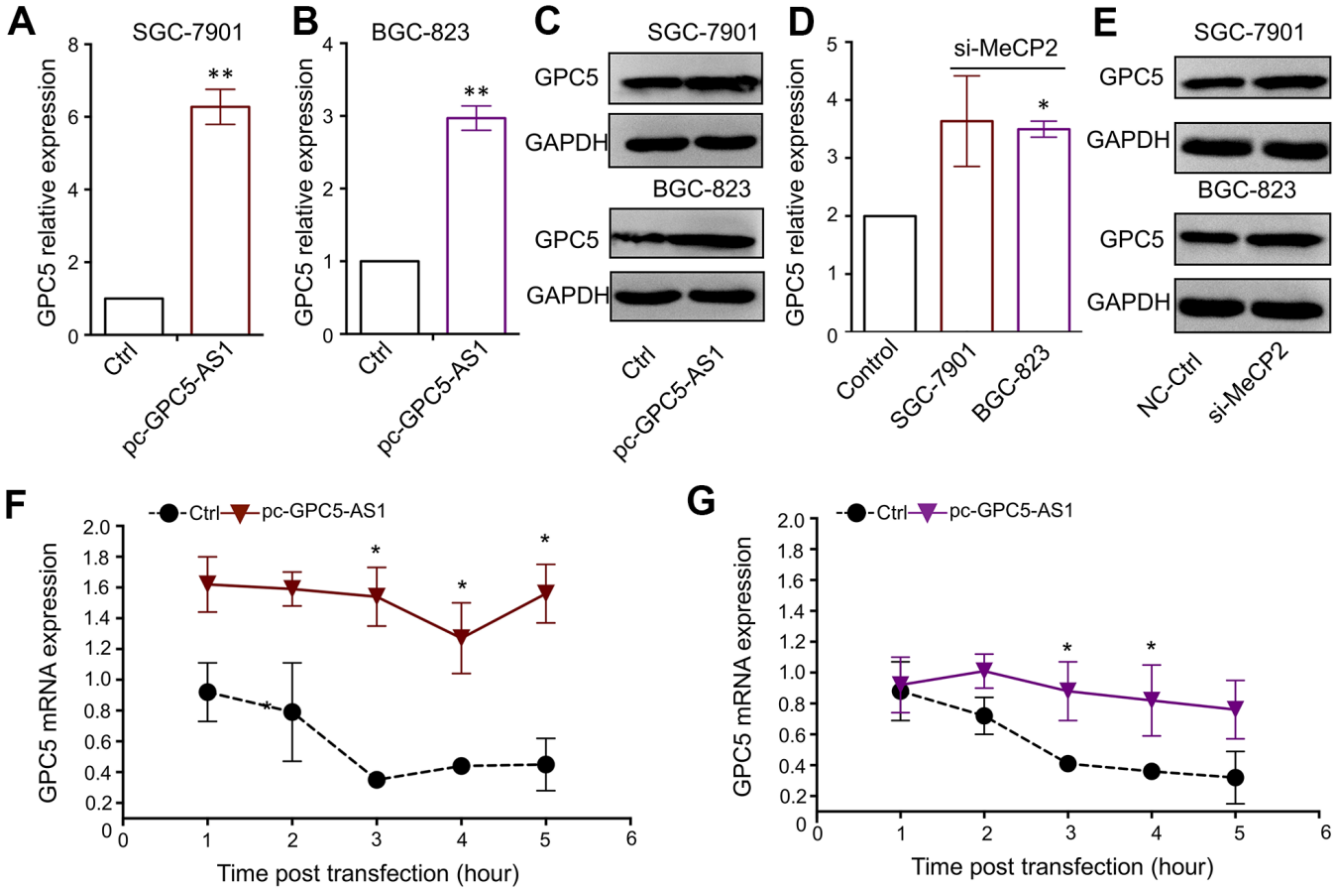
**GPC5-AS1 regulates GPC5 expression by enhancing mRNA stability.** (**A**, **B**) GPC5 mRNA expression was measured in SGC-7901 and BGC-823 cells transfected with pc-GPC5-AS1. (**C**) GPC5 protein expression was measured in SGC-7901 and BGC-823 cells transfected with pc-GPC5-AS1. (**D**, **E**) GPC5 mRNA and protein expression was measured in SGC-7901 and BGC-823 cells transfected with si-MeCP2. (**F**, **G**) GPC5 mRNA expression level was measured at a time curve in SGC-7901 and BGC-823 cells transfected with pc-GPC5-AS1 or ctrl, following treatment with actinomycin D. (p* < 0.05, p** < 0.01).

Both GPC5-AS1 and GPC5 were found to be downregulated in GC cells and suppress GC progression. The qRT-PCR and western blotting analyses confirmed the remarkable positive regulation of GPC5 expression after GPC5-AS1 acceleration (Figure 5A–5C). Moreover, the silencing of MeCP2 also resulted in a slight increase in GPC5 expression in BGC-823 and SGC-7901 cells (Figure 5D, 5E). LncRNA have been reported to mediate target gene expression by stabilizing the transcripts, and our results showed that following treatment with actinomycin D, GPC5-AS1 overexpression led to a deferred degradation of GPC5 mRNA (Figure 5F, 5G).

### GPC5-AS1 sequesters members of the miR-106a family in GC cells

**Figure 6:**
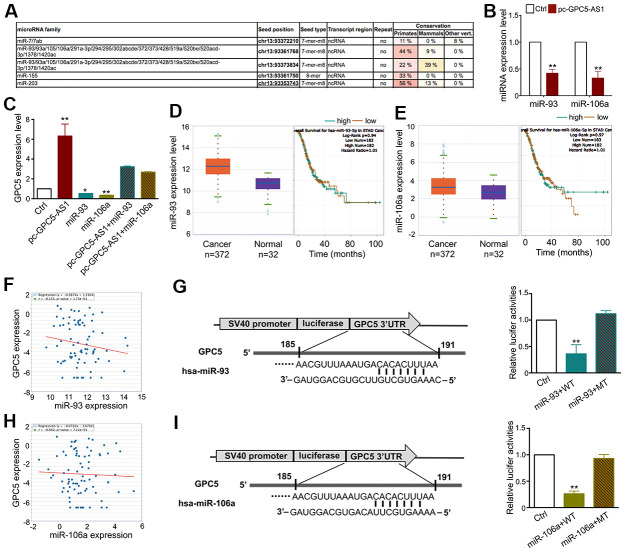
**GPC5-AS1 functions as a sponge of the miR-106a family in GC cells.** (**A**) The results from the miRcode database revealed that GPC5-AS1 has binding sites with the miR-106a family. (**B**) As represented, miR-93 and miR-106a expression levels were measured by qRT-PCR in SGC-7901 cells transfected with pc-GPC5-AS1. (**C**) GPC5 expression was analyzed in SGC-7901 cells with pc-GPC5-AS1, miR-93 or miR-106a mimics alone or together. (**D**, **E**) Expression levels of miR-93 and miR-106a in GC according to the TCGA database. (**F**) Correlation analysis of GPC5 and miR-93 expression in GC tissues through starBase. (**G**) The bioinformatics analysis of miR-93 targeting GPC5 and this relationship was validated by dual-luciferase reporter assay in HEK-293 cells. (**H**, **I**) Interaction between miR-106a and GPC5 was verified by bioinformatics and dual-luciferase reporter analysis. (p* < 0.05, p** < 0.01).

Studies have demonstrated that lncRNAs can function as ceRNAs by binding with several miRNAs and regulate their target gene expression. To clarify whether GPC5-AS1 regulates GC progression as a ceRNA, we utilized the bioinformatics database miRcode to predict the top negatively correlated miRNAs (miR-93 and miR-106a), which were also predicted to directly target the GPC5 3′-UTR region (Figure 6A). Furthermore, qRT-PCR was performed to investigate the inverse correlation of miR-93 and miR-106a with GPC5-AS1 in SGC-7901 cells (Figure 6B). Subsequently, we measured the expression of GPC5 after transfecting with miR-93 or miR-106a mimics in the presence or absence of pc-GPC5-AS1, and found that GPC5-AS1 overexpression could fully rescue miR-93 and miR-106a induced GPC5 mRNA inhibition (Figure 6C). TCGA database revealed that miR-93a and miR-106 were downregulated (Figure 6D, 6E) and negatively correlated with GPC5 in GC samples (Figure 6F, 6H). As miRbase predicted, miR-93 and miR-106a may bind to the 185–191 bp site in the 3′-UTR of GPC5. To verify this prediction, we used a dual-luciferase reporter assay in HEK-293 cells. miR-93 or miR-106a mimics reduced the luciferase signals of GPC5-WT cells significantly, and had no effect on the luciferase activity of GPC5-MT (Figure 6G, 6I). Considering these findings, GPC5-AS1 appears to be acting as a molecular sponge for miR-93 and miR-106a in GC.

## DISCUSSION

Previously, we analyzed MeCP2-associated ChIP-Seq data and identified GPC5-AS1 as a novel lncRNA. The current study demonstrated that GPC5-AS1 is significantly down-regulated in GC cells and tissues compared with that in adjacent normal tissues. MeCP2 has been proven to act as an epigenetic factor and regulate gene transcription through binding to methylated CpG islands [20]. Herein, our ChIP RT-PCR and luciferase reporter analysis results verified that GPC5-AS1 is a gene directly targeted by MeCP2. Specifically, it was revealed that MeCP2 binds with GPC5-AS1 on the methylated CpG sites in the promoter regions, and MeCP2 silencing induced a significant increase in GPC5-AS1 expression in GC cells.

LncRNAs play a crucial role in the regulation of gene expression, and progression of cancer. Recent bioinformatic analyses indicate that dysregulated expression of GPC5-AS1 is associated with several tumors, such as metastatic melanoma [21], renal cell cancer [22, 23], astrocytoma [24], and meningioma [25]. GPC5-AS1 is considered to play a prominent role in tumor progression. Nevertheless, the function of GPC5-AS1 in GC is unknown. In the present study, gain-of-function and loss-of-function experiments revealed that accelerated GPC5-AS1 expression suppressed GC cell growth, cell cycle transition, colony forming capacity, and induced cell apoptosis *in vitro*. Similarly, silencing GPC5-AS1 slightly promoted tumor formation *in vivo*. Furthermore, a xenograft model study indicated that GPC5-AS1 could remarkably inhibit the tumorigenicity of GC cells *in vivo*. These data conclude that GPC5-AS1 may have an antitumor effect in GC progression. However, the molecular mechanism of its antitumor effect in GC requires further investigation.

A widely accepted regulatory pattern of the role lncRNAs play in cancer is to competitively sponge miRNAs and preserve their target mRNAs. MT1JP, a downregulated lncRNA associated with the survival of patients with GC, regulates the progression of GC as a ceRNA, by competitively binding to miR-92a-3p and regulating FBXW7 expression [18]. Meanwhile, the overexpressed lncRNA PVT1 can elevate epithelial mesenchymal transition (EMT) marker levels, and promote EMT processes and tumor metastasis of GC *in vitro* and *in vivo*, by binding to miR-30a and increasing its target gene Snail expression by acting as a competing endogenous RNA [26]. Using bioinformatic tools, we predicted that GPC5-AS1 may be a potential target for the miR-106 family. As representative members, miR-93 and miR-106a are known to play key roles in GC genesis. miR-93 was found to be overexpressed and facilitated the development of GC cell growth *in vitro* and in xenograft mice by negatively regulating PDCD4 [27]. miR-106a was over-expressed in GC tissues, in comparison with their matched normal counterparts and could inhibit cell apoptosis by interfering with the FAS-mediated apoptotic pathway [28]. Our experiments validated the inverse correlation of miR-93 and miR-106a with GPC5-AS1 in GC cells.

GPC5, the host gene of GPC5-AS1, is also predicted to have binding sites in 3′-UTR for miR-93 and miR-106a. GPC5 is a member of heparan sulfate proteoglycans family and firstly known as a key regulator of growth factors and morphogens [29, 30]. Recent reports have revealed its relevance to the tumorigenic process in various types of cancers. In prostate cancer, GPC5 is downregulated, and its upregulation significantly inhibits cancer cell growth and cell invasion by targeting Sp1 through EMT inhibition and Wnt/β-catenin signaling activation [31]. In hepatocellular carcinoma (HCC), GPC5 has been revealed to be a direct target of miR-709, and enforced expression of GPC5 inhibited HCC cell proliferation and invasion [32]. However, the biological importance of GPC5 in the initiation and progression of GC remains controversial. GPC5 was verified as a new oncogene, which could promote the proliferation and invasion of GC cells [33]; however, in another study, it was not expressed in any of the GC tissue samples, as determined using qPCR [34]. In our study, GPC5 was experimentally verified to be downregulated in GC tissues, and elevated GPC5 could restrain GC cell growth and promote cell apoptosis. TCGA database revealed that miR-93a and miR-106 were downregulated and negatively correlated with GPC5 in GC samples. Our dual-luciferase reporter data verified the direct correlation between GPC5 and miR-93 or miR-106a. Furthermore, GPC5-AS1 overexpression fully rescued miR-93- and miR-106a- induced GPC5 mRNA inhibition.

To summarize, we revealed that a novel lncRNA, GPC5-AS1, is downregulated in GC, and this aberrant expression is regulated by MeCP2, which binds to the CpG site in the GPC5-AS1 promoter region. Increased GPC5-AS1 level was observed to inhibit GC cell proliferation both *in vitro* and *in vivo*. Regarding the molecular mechanism of anti-tumor action, we reported that GPC5-AS1 can stabilize GPC5 mRNA as the molecular sponge of miR-93 and miR-106a. Our findings indicate that GPC5-AS1 holds potential to be a novel diagnostic biomarker and a promising target for GC therapeutic strategies, providing a theoretical foundation for the development of treatments for GC.

## MATERIALS AND METHODS

### Clinical samples

Human GC tumors and matched non-tumor tissues (5 cm away from tumor border) were collected from 53 GC patients at the First Affiliated Hospital of Xi’an Jiaotong University from 2012 to 2014. Informed consent was obtained from each patient before the surgical resection, and approval for this study was granted by the Institute Research Ethical Committee at the Cancer Center of Xi’an Jiaotong University, and the guidelines of the committee were followed.

### Cell culture

Human GC cell lines, gastric epithelial cell lines, and embryonic kidney cell lines were obtained from the Genechem Cell Bank (Shanghai, China). Cells were cultured in RPMI-1640 medium (Biological Industries, Beit HaEmek, Israel) containing 10% fetal bovine serum (Biological Industries) with 1% penicillin/streptomycin (Solarbio, Beijing, China) at 37° C in 5% CO_2_ incubator.

### Immunohistochemistry (IHC)

Formalin-fixed paraffin-embedded GC tissues were sectioned and deparaffinized with xylene and hydrated using graded alcohol. After antigen retrieval and blocking, slides were incubated with anti-GPC5 antibody and matched secondary antibody, followed by 3, 3’-diaminobenzidine (DAB), and hematoxylin staining. Then the intensity was manually scored, and a sample with a percentage of positive cells of more than 50% in five randomly selected fields was considered highly expressed.

### RNA isolation and quantitative real-time PCR (qRT-PCR)

Total RNA from cell lines or frozen tissues and FFPE tissue samples was extracted using TRIzol Reagent (Invitrogen, CA, USA) and Qiagen FFPE RNeasy Kit following the manufacturer’s protocols, respectively. The concentration of each RNA sample was measured using Nanodrop (Thermo Fisher, Wilmington, USA) for cDNA synthetization, and qRT-PCR was performed with SYBR Green PCR kit (GenStar, Beijing, China). All PCR reactions were executed in triplicate using IQ5 qRT-PCR Detection platform (Bio-Rad, CA, USA). β-Actin and U6 were considered house-keeping genes for mRNA and miRNA, respectively. The 2^−ΔΔCt^ method was performed in the qRT-PCR analysis. All primer sequences used for PCR are listed in Supplementary Table 1.

### Western blot

Total proteins of each sample were extracted with RIPA lysis reagents containing phosphatase and protease inhibitors (Beyotime, Beijing, China). Equal amounts of protein were separated with SDS-PAGE gels and transferred to PVDF membranes. The membranes were blocked with 5% fat-free milk in Tris-buffered saline Tween-20 buffer at room temperature and incubated with different primary antibodies and the matched secondary antibodies. After that, the membranes were covered with ECL solution (Pierce, IL, USA) for chemiluminescence measurement through Syngene GBox (Syngene, Cambridge, UK). The antibodies used for the differentially expressed gene are listed in Supplementary Table 2.

### Plasmid construction, siRNA synthesis, and transfection

The short-hairpin RNA (sh-RNA) targeting GPC5-AS1 and GPC5 were purchased from the company (Genechem). siRNA was pre-designed and chemically synthesized for the silence of MeCP2 gene expression (GenePharma, Shanghai, China) and non-sense shRNA or siRNA was considered a negative control. The plasmid or siRNA was transfected using Lipofectamine-2000 (Invitrogen) according to the manufacturer’s protocol. The sequence of siRNA or shRNA used is listed in Supplementary Table 3.

The MeCP2 plasmid (WT), Mutation type 1 plasmid (MT1), Mutation type 2 plasmid (MT2), and reporter plasmid pGL3-GPC5-AS1 were constructed as our previous study described [11]. The sequence of MeCP2 plasmid construction is listed in Supplementary Tables 4, 5. GPC5-AS1 expression plasmid was purchased and verified by Suzhou Genewiz. MiR-93 and miR-106a vectors were constructed by using the pcDNA^™^6.2-GW/EmGFP-miR vector (Invitrogen).

### Chromatin immunoprecipitation (ChIP), ChIP-Seq and ChIP-qRT-PCR

Cells were cross-linked and nuclear lysates were sonicated for ChIP-Seq analysis has been described in our previous study [11]. Meanwhile, DNA from chromatin lysates incubated with anti-MeCP2 or IgG antibody was extracted using the QIA quick PCR purification kit (Qiagen, CA, USA) for ChIP-qRT-PCR experiments. The sequences of primer used for ChIP-qRT-PCR are listed in Supplementary Table 1.

### Cell viability assay

Cells were seeded into 96-well plates and cell viability was performed with MTT assays (Sigma, MO, USA) every 24 hours after transfection. After 72 hours, 10 μl of MTT regents was added and incubated for 4 h at 37° C. Then the supernatants were removed and precipitations were dissolved with 150 μl dimethylsulfoxide (DMSO). Absorbance was detected with POLARstar microplate reader (BMG Labtech GmbH, Ortenberg, Germany) at 492 nm wavelength.

### Cell cycle analysis

Cells were harvested at 48 h after transfection and washed twice with ice-cold PBS and stored at 4° C overnight within 70% ethanol. Before the cell cycle analysis, cells were incubated at room temperature with 0.1 mg/ml RNase A and 0.05 mg/ml propidium iodide (PI) for 15 min, then the population of differential stages was measured by flow cytometry (BD, CA, USA).

### Cell apoptosis analysis

Cells were harvested at 24 h after transfection and stained by using the Annexin V-FITC/PI Apoptosis Detection kit (Invitrogen) following the manufacturer’s protocol. The population of apoptotic cells was quantified with flow cytometry (BD).

### Tumorigenicity analysis in nude mice

Four-week-old male BALB/c nude mice bred under specific pathogen-free conditions in the Central laboratory of animals of Xi’an Jiaotong University were used to examine tumorigenicity. 1×10^6^ cells transfected with lv-GPC5-AS1 or lv-ctrl (Genechem) were resuspended in 100 μl PBS and injected subcutaneously into two-side flanks of the mice. Tumor volume was measured and calculated using vernier calipers with following the formula: tumor volume = (length × width^2^) / 2 once every 3 days. On day 28, after euthanizing the mice through cervical dislocation, the tumors were removed and weighed.

### Actinomycin D assay

Cells after transfection were treated with 5 μmol/L Actinomycin D for a specific time dose before RNA extraction with TRIzol reagent. Then, the change in RNA levels was analyzed by using qRT-PCR.

### Dual-luciferase assay

The miR-93 or miR-106a expression vector was co-transfected with GPC5-WT or GPC5-MT vectors, and pmir-GLO and pcDNATM6.2 vectors were co-transfected as control into HEK-293 cells at 96-well plate. The fluorescence expression was analyzed using a Dual-Luciferase Reporter assay kit (Promega, WI, USA) within 24 hours following the manufacturer’s protocols.

### Statistical analysis

All experiments were carried out three times unless otherwise stated. Statistical analyses were performed with GraphPad Prism and Student’s *t*-test was used to estimate the difference between two independent groups. Mean ± SD are presented and *p* values < 0.05 were considered to be significant differences.

### Ethics statement

This study was approved by the Institutional Animal Care and Use Committee of Xi’an Jiaotong University. Written informed consent was obtained from each patient.

## Supplementary Material

Supplementary Tables
