# Supplementary material for: The novel lncRNA GPC5-AS1 stabilizes GPC5 mRNA by competitively binding with miR-93/106a to suppress gastric cancer cell proliferation
Source: Aging (Albany NY). 2022 Feb 18;14(4):1767–81. doi: 10.18632/aging.203901 (PMC8908922; doi:10.18632/aging.203901)
Supplement: Supplementary Tables [file aging-14-203901-s001.pdf]

SUPPLEMENTARY TABLES

Supplementary Table 1. Primer sequences used for qRT-PCR in this study.

Gene	Sequence
GPC5-AS1-F	5'-AGAAGAGGGAAAGGGAAGGTC-3'
GPC5-AS1-R	5'-ACTACTGGGGAGGCTGAGGT-3'
GPC5-F	5'-TGTGATGATGAAGATGGTTGC-3'
GPC5-R	5'-GCCAGTGTCTGTTTGATGGA-3'
GPC5-AS1 promoter region-F	5'-CGGACATTTGGGGTATTA-3'
GPC5-AS1 promoter region-R	5'-TCGCAGGCATTAGGTGGT-3'
GAPDH-F	5'-TGAAGGTCGGAGTCAACGGATT-3'
GAPDH-R	5'-CCTGGAAGATGGTGATGGGATT-3'
hsa-miR-93-RT	5'-GTCGTATCCAGTGCGTGTCGTGGAGTCGGCAATTGCACTGGATACGACCTACCTG-3'
hsa-miR-93-F	5'-ATCCAGTGCGTGTCGTG-3'
hsa-miR-93-R	5'-TGCTCAAAGTGCTGTTCGTG-3'
hsa-miR-106a-RT	5'-GTCGTATCCAGTGCGTGTCGTGGAGTCGGCAATTGCACTGGATACGACCTACCTG-3'
hsa-miR-106a-F	5'-ATCCAGTGCGTGTCGTG-3'
hsa-miR-106a-R	5'-TGCTAAAAGTGCTTACAGTG-3'
U6-RT	5'-CGCTTCACGAATTTGCGTGTCAT-3'
U6-F	5'-GCTTCGGCAGCACATATACTAAAAT-3'
U6-R	5'-CGCTTCACGAATTTGCGTGTCAT-3'

Supplementary Table 2. Information on antibodies used for the correlation analysis.

Antibody	WB	Specificity	Company
MeCP2 (sc-20700)	1:1000	Rabbit polyclonal	Santa Cruz Biotechnology
GPC5 (ab123886)	1:1000	Rabbit Monoclonal	Abcam
GAPDH (sc-8432)	1:1000	Mouse Monoclonal	Cell Signaling Technology
MeCP2 ChIP Grade (ab2828)	-	Rabbit polyclonal	Abcam
GFP ChIP Grade (ab290)	-	Rabbit polyclonal	Abcam
IgG ChIP Grade (ab171870)	-	Rabbit polyclonal	Abcam

Supplementary Table 3. Sequences of siRNA or shRNA.

Name	Sequence
negative siRNA (NC-siRNA) sense	5′-UUCUCCGAACGUGUCACGUTT-3′
negative siRNA (NC-siRNA) antisense	5′- ACGUGACACGUUCGGAGAATT-3′
si-MeCP2 sense	5′-GCUUAAGCAAAGGAAAUCUTT-3′
si-MeCP2 antisense	5′-AGAUUUCCUUUGCUUAAGCTT-3′
sh-Control target sequence	5'-TTCTCCGAACGTGTCACGT-3'
sh-GPC5-AS1 target sequence	5'-GCAACTCCACATAATATCTGG-3'
sh-GPC5 target sequence	5'-GCATGGAACATACGACATTGG-3'

Supplementary Table 4. Sequences of MECP2 MT recombinant plasmids.

Name	Sequence
MECP2-WT MBD sequence	5'-AGGGGCCCTATGTATGATGACCCTACACTGCCCGAGGGCTGGACCAGGAAACTGAA GCAGAGGAAGTCCGGA**AGGAGCGCCGGCAAATACGAT**GTCTACCTGATTAACCCCCA GGGCAAGGCCTTTAGATCCAAGGTGGAGCTG**ATCGCCTACTTTGAGAAGGTC**GGCGA CACATCCCTAGACCCGAATGACTTCGACTTCACAGTGACCGGCAGAGGA-3'
MECP2-MT1 MBD sequence	5'-AGGGGCCCTATGTATGATGACCCTACACTGCCCGAGGGCTGGACCAGGAAACTGAA GCAGAGGAAGTCCGGA**GATCAGTATCACGAAGGTCGC**GTCTACCTGATTAACCCCCA GGGCAAGGCCTTTAGATCCAAGGTGGAGCTGATCGCCTACTTTGAGAAGGTCGGCGAC ACATCCCTAGACCCGAATGACTTCGACTTCACAGTGACCGGCAGAGGA-3'
MECP2-MT2 MBD sequence	5'-AGGGGCCCTATGTATGATGACCCTACACTGCCCGAGGGCTGGACCAGGAAACTGAA GCAGAGGAAGTCCGGAAGGAGCGCCGGCAAATACGATGTCTACCTGATTAACCCCCAG GGCAAGGCCTTTAGATCCAAGGTGGAGCTG**CATGACAAGCAGGCCGAGAGT**GGCGA CACATCCCTAGACCCGAATGACTTCGACTTCACAGTGACCGGCAGAGGA-3'

Supplementary Table 5. Sequences of MeCP2 binding to GPC5-AS1 promoter region.

Gene	Sequence
GPC5-AS1	ATCTATTTATTGTCTTTAAGTTGGACATCTTCCTCTCCAACTTAGTTTAATGA GTGGTTTTCAGCTGAAACCCGGACATTTGGGGTATTATGTGTAAGGGACTCC AGATATTATTTCAACCTTCCGTTTTAGCTGATTTCCACAGACTCCATTACTGC AGGCTATGGGAGAGCACCACCTAATGCCTGCGAGGTGTGGATAAAAGTCTG
